# CRISPR/Cas9-mediated correction of mutated copper transporter ATP7B

**DOI:** 10.1371/journal.pone.0239411

**Published:** 2020-09-30

**Authors:** Michael Pöhler, Sarah Guttmann, Oksana Nadzemova, Malte Lenders, Eva Brand, Andree Zibert, Hartmut H. Schmidt, Vanessa Sandfort

**Affiliations:** 1 Medizinische Klinik B, Gastroenterologie, Hepatologie, Endokrinologie, Klinische Infektiologie, Universitätsklinikum Münster, Münster, Germany; 2 Medizinische Klinik D, Allgemeine Innere Medizin und Notaufnahme sowie Nieren- und Hochdruckkrankheiten und Rheumatologie, Universitätsklinikum Münster, Münster, Germany; University of Saskatchewan, CANADA

## Abstract

Wilson's disease (WD) is a monogenetic liver disease that is based on a mutation of the *ATP7B* gene and leads to a functional deterioration in copper (Cu) excretion in the liver. The excess Cu accumulates in various organs such as the liver and brain. WD patients show clinical heterogeneity, which can range from acute or chronic liver failure to neurological symptoms. The course of the disease can be improved by a life-long treatment with zinc or chelators such as D-penicillamine in a majority of patients, but serious side effects have been observed in a significant portion of patients, e.g. neurological deterioration and nephrotoxicity, so that a liver transplant would be inevitable. An alternative therapy option would be the genetic correction of the *ATP7B* gene. The novel gene therapy method CRISPR/Cas9, which has recently been used in the clinic, may represent a suitable therapeutic opportunity. In this study, we first initiated an artificial *ATP7B* point mutation in a human cell line using CRISPR/Cas9 gene editing, and corrected this mutation by the additional use of single-stranded oligo DNA nucleotides (ssODNs), simulating a gene correction of a WD point mutation *in vitro*. By the addition of 0.5 mM of Cu three days after lipofection, a high yield of CRISPR/Cas9-mediated *ATP7B* repaired cell clones was achieved (60%). Moreover, the repair efficiency was enhanced using ssODNs that incorporated three blocking mutations. The repaired cell clones showed a high resistance to Cu after exposure to increasing Cu concentrations. Our findings indicate that CRISPR/Cas9-mediated correction of *ATP7B* point mutations is feasible and may have the potential to be transferred to the clinic.

## Introduction

The genome editing tool CRISPR/Cas9 (clustered regularly interspaced short palindromic repeats (CRISPR) associated nuclease 9) offers a new gene therapeutic potential to efficiently target inherited monogenetic or infectious diseases. Within the last couple of years it has been used to correct the genetic basis of many diseases in animal models or isolated cells [1–6]. WD is an excellent model to study genetic corrections, since a majority of WD patients carry point mutations such as H1069Q, which is the most frequent mutation in the Caucasian population [7]. This inherited autosomal recessive disorder is caused by mutations in the *ATP7B* gene encoding for a copper (Cu) efflux pump [8]. It provokes a functional impairment of Cu excretion by the liver, followed by excess Cu deposition in organs, mostly in the liver and brain [9]. Patients display clinical heterogeneity ranging from acute or chronic liver failure and/or neurological symptoms [10]. The progression of WD can be partly ameliorated by zinc or chelating agents such as D-penicillamine and trientine [11–13]. Though these treatments are usually effective, severe side effects have been reported in a significant portion of WD patients [14, 15]. As a result, patients may stop the medication, leading to an acute clinical presentation with rapid deterioration [10, 16]. The only curative therapy remains an orthotropic liver transplantation [17]. Gene therapy may overcome the need for liver transplantation as well as the shortage of donor livers [18]. The traditional approach of gene therapy is to transfer a functional copy of the mutated gene within clinically relevant cells from the patient using viral vectors. For inherited metabolic diseases of the liver, the goal is to obtain high expression levels in the patient’s hepatocytes correcting the disease phenotype. However, the risk of a gene therapy approach is that viral DNA may being incorporated randomly into cellular DNA, disrupting a valuable gene such as a tumor-suppressor gene [19]. Other genome-editing technologies have been widely used to modify or inactivate specific genes in therapeutic approaches or in functional studies, such as zinc finger nucleases (ZFNs) and transcription activator-like effector nucleases (TALENs) [20, 21]. In 2013, the CRISPR/Cas9 system has become a powerful gene editing tool and replaced the previously developed technologies [22–24]. In this system, a single guide RNA (sgRNA) is used to guide the Cas9 nuclease to target DNA containing the protospacer adjacent motif (PAM), which is 5´- NGG -3´ for *Streptococcus pyogenes* Cas9. A double-strand break (DSB) is generated by Cas9 at ∼3 base pairs upstream from the PAM region. Two major repair mechanisms may be activated after a DSB. The error-prone non-homologous end joining (NHEJ) results in a variety of mutations, such as insertion/deletion (INDEL) frameshift mutations leading to transcript degradation [25]. Thus, NHEJ is used intentionally in order to initiate a gene knockout (KO). The second repair mechanism occurs during homologous recombination, represented by the homology-directed repair (HDR) [23, 25], which repairs a DSB precisely using a DNA repair template. The HDR mechanism can only be utilized by the cell in the presence of a homologous set of DNA, usually the sister chromatid, within the G2 stage of the cell cycle. As a tool for site-specific single base corrections, the introduction of a single-stranded oligo DNA nucleotide (ssODN) into a target cell may lead to the repair of an aberrant gene after DSB [26, 27]. The design of ssODNs as well as the directed shift from the NHEJ to the HDR pathway has been improved to enhance the efficiency of gene modification [28, 29]. To prevent a re-editing by the highly active Cas9 nuclease, the introduction of blocking mutations in ssODNs located within the PAM sequence or guide RNA target have minimized undesirable re-editing during gene editing [30].

CRISPR/Cas9 technology has been applied in two studies targeting WD. Jiang et al. created a single amino acid substitute rabbit model for WD, representing the most frequent WD missense mutation in Asia (p. Arg778Leu) in exon 8 of *ATP7B* [31, 32]. Lately, Liu et al. replaced exon 8 of the *ATP7B* gene in a mouse model using CRISPR/Cas9 [33]. However, a gene correction of *ATP7B* point mutations on human cellular level has not been described so far.

Prior studies by our lab established a novel *ATP7B* KO human intestinal cell line (Caco-2 cells) using CRISPR/Cas9 technology, demonstrating a crucial role of Cu and *ATP7B* in the storage, processing, and secretion of lipids in a human enterocyte model [34]. In the present study, our aim was to create a point mutation in order to mimic a WD-specific mutation leading to a loss of function of the *ATP7B* gene. Subsequently, the initiated point mutation within the *ATP7B* gene was repaired using the CRISPR/Cas9 system plus specific ssODNs, with focus on cell selection efficiency by Cu addition. Since WD is characterized by the dysfunction of the Cu transporting protein ATPase7B, genetically corrected cells can be positively selected *in vitro* by the addition of Cu [35].

## Materials and methods

### Cell culture

HEK293T cells were purchased from German tissue culture collection (DSMZ, # ACC 635) and cultivated under 5% CO_2_ at 37°C in a humidified chamber. The cells were cultured in Dulbecco’s Modified Eagle’s Medium (DMEM) High Glucose with L-glutamine (GE Healthcare, Chicago, IL, USA) supplemented with 10% fetal bovine serum (Gibco, Carlsbad, CA, USA) and 100 U/ml penicillin/streptomycin (Hyclone, Logan, UT, USA).

### sgRNA design

The sgRNA was designed using the CRISPR/Cas9 Design Tool (http://crispr.mit.edu) to minimize potential off-target effects [36], targeting exon 2 of the *ATP7B* gene. The sgRNA sequence (5’-ATATCGGTGTCTTTGGCCGA-3’) was inserted via *BbsI* into the pSpCas9(BB)-2A-Puro (PX459) V2.0 plasmid (Addgene #62988), which was a gift from Feng Zhang [37], named PX459.ATP7B.

### Mutagenesis

For generation of the PX459.ATP7BΔC plasmid, a site-directed mutagenesis was performed. The PX459.ATP7B plasmid was modified using QuikChange II XL Site-Directed Mutagenesis Kit (Agilent Technologies) [38]. The primer sequences were: (5′-3′): TTCTAGCTCTAAAACTCGCCAAAGACACCGATATCG and (5′-3′): CGATATCGGTGTCTTTGGCGAGTTTTAGAGCTAGAA.

### ssODN repair template design

A total of 12 ssODN repair templates were designed with homologous genomic flanking sequences varying in nucleotide (NT) arm length (30, 40, 50 or 60 NT´s) centered around the targeted CRISPR/Cas9 cleavage site. ssODN repair templates contained 2 to 3 silent CRISPR/Cas9 blocking mutations (ssODN_2M, ssODN_3M) or no blocking mutation (ssODN_C) (PAGEpurified, IDT, Coralville, IA, USA; Fig 3 and S1 Table).

### Cell transfection

For CRISPR/Cas9-mediated *ATP7B* KO experiments, 0.5 x 10^6^ HEK293T cells were seeded in one well of a 6-well plate using standard cell culture medium. The next day, 2 μg of the targeting vector PX459.ATP7B was transfected into HEK293T cells with Lipofectamine 2000 (Invitrogen, Carlsbad, CA, USA) according to manufacturer instructions. After 24 hours, cells were seeded as single cells in 96-well plates and selected with 1 μg/ml puromycin. After 72 hours, medium was changed to standard cell culture medium. Single‐cell‐derived clonal cell lines were obtained after 2 to 3 weeks for further analysis.

For CRISPR/Cas9-mediated *ATP7B* repair experiments, 0.5 x 10^6^ HEK293TΔC cells were lipofected (Lipofectamine 2000) with 1 μg of PX459.ATP7BΔC plasmid plus a mixture of four different ssODNs (0.25 μg each), varying in total length (61, 81, 101 and 121 nt) (S1 Table). One group received four different ssODNs exhibiting three blocking mutations (ssODN_3M), another group received four ssODNS with two blocking mutations (ssODN_2M) and the last group was transfected with four ssODNs carrying no blocking mutations as a control (ssODN_C). Cells were positively selected with 0.5 mM of copper chloride (CuCl_2,_ Sigma Aldrich, St. Louis, MO, USA) in standard culture medium at 24 hours (day 1) or 72 hours (day 3) after lipofection for two days before medium was changed to standard culture medium. Two to three weeks after lipofection cells were plated to 96-well plates for single-cell-cloning.

After cultivation in 96-well plates for 15 days, the monoclonal cells were transferred in 6-well plates for 6 days. Chromosomal DNA of selected cell clones was isolated using QIAamp DNA mini kit (Qiagen), followed by Sanger sequencing using primers 5’-AGAGGGCTATCGAGGCAC-3’ / 5’-GGGCTCACCTATACCACCATC-3’ and Big Dye Version 3.1 (Life Technologies) to confirm editing efficiency.

### MTT assay

Cells were seeded in triplicates in 96-well plates (Corning, Corning, NY, USA) and cultivated in phenol red free cell culture medium (Lonza). Subsequently, cells were treated with Cu concentrations for 48 hours using different concentrations of CuCl_2_. Next, MTT (1mg/ml 3-[4, 5-dimethylthiazolyl-2]-2, 5-diphenyltetrazolium bromide; Sigma-Aldrich) was added for 2 hours and solubilized with sodium dodecyl sulfate (SDS; Roth, Karlsruhe, Germany) and dimethyl sulfoxide (DMSO; Roth) to determine cell viability. Absorbance was measured at 560 nm and viability was calculated as percentage of untreated control cells (100%).

### Real-time quantitative PCR

The RNeasy kit (Qiagen, Hilden, Germany) was used to isolate total RNA and transcription was performed using 1 μg of RNA and SuperScript III (Invitrogen, Carlsbad, CA, USA). Rox SYBR Master Mix (Eurogentec, Liège, Belgium) was used to perform real-time qPCR analysis. Ct values were normalized to the expression of the *GAPDH* house-keeping gene (ΔΔCt method) and log2 expression was calculated. PCR analysis was carried out on the CFX384 Touch Real-Time PCR Detection System (Bio-Rad Laboratories, Inc., Hercules, USA). The following primer sequences were used: *ATP7A* (forward/reverse, 5′-3′): AGCAATGGCTGCTTCATCTG/GCAGGCAGTTCATAACTCTCG and *GAPDH* (forward/reverse, 5′-3′): CCCACTCCTCCACCTTTGAC/ CCACCACCCTGTTCCTGTAG.

### Western Blot

Cells were homogenized in RIPA lysis buffer (60 mM tris-HCl, 150 mM NaCl, 2% Na-deoxycholate, 2% Triton X-10, 0.2% SDS, and 15 mM EDTA) and protease inhibitors (Roche, Basel, Switzerland; Complete Mini, EDTA-free). 10 μg protein lysate of whole protein extract was separated on a 10% SDS gel. Samples were blotted onto PVDF membranes and blocked with 5% semi-skimmed milk powder (Roth, Karlsruhe, Germany) in Tris-buffered saline supplemented with 0.1% Tween 20 (Merck, Darmstadt, Germany). For detection of ATP7B protein (ATPase7), a monoclonal rabbit anti-human ATP7B antibody (1:1,000, ab124973, Abcam, Cambridge, UK) was added overnight. β-Actin was assessed for protein loading control (1:1,000, sc-47778 HRP, Santa Cruz Biotechnology, Santa Cruz, CA, USA). Secondary antibody was horseradish peroxidase-conjugated anti-rabbit (1:10,000; GE Healthcare life science, München, Germany) and incubated 2 hours at room temperature. For detection, ECL Western Blotting Detection Reagent (GE Healthcare) was added and the membrane was exposed to film (Hyperfilm, GE Healthcare).

### Statistical analysis

SPSS 24.0 software (IBM Armonk, NY, USA) was used for statistical analysis. Data were analyzed by the Kruskal-Wallis test or the Wilcoxon-Mann-Whitney test. *P* values < 0.5 were considered as significant and data are given as mean ± SEM.

## Results

### Generation of *ATP7B* knockout by CRISPR/Cas9

**Fig 1:**
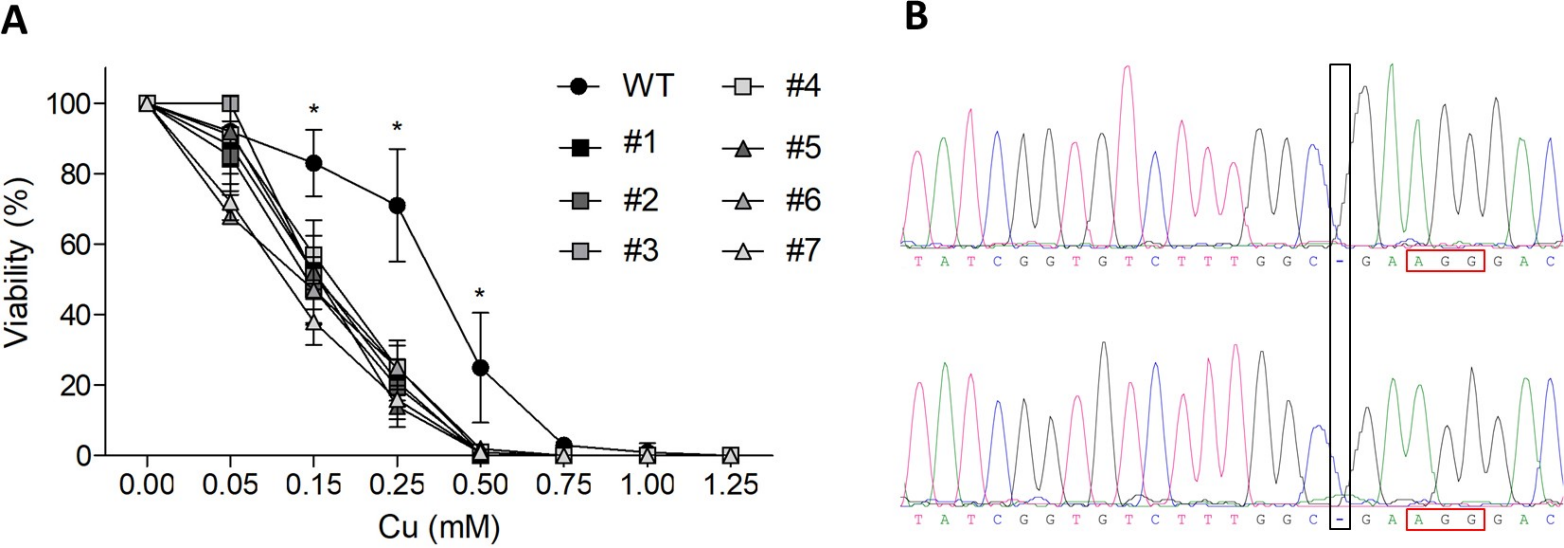
CRISPR/Cas9-mediated *ATP7B* KO reduced Cu resistance. **(A)** HEK293T clonal cells lipofected with PX459.ATP7B vector were cultivated for 48 hours using cell culture medium containing the indicated concentrations of Cu. Cellular metabolic activities of cells were determined by MTT assay. HEK293T *ATP7B* WT cells served as control (circle). Four *ATP7B* homozygous (rectangle), and three heterozygous (triangle) cell clones are shown. The survival of cells is given as percentage relative to cells that received no Cu treatment (100%). Mean ± SE of four experiments is shown. * indicate significance (p < 0.05). **(B)** Sanger sequencing of HEK293T cell clone #1 after CRISPR/Cas9-mediated *ATP7B* KO revealed a deletion of one cytosine nucleotide at position 1184 (black rectangle), three nucleotides upstream the PAM region (red rectangle). Forward (top) and reverse (bottom) sequence analysis is depicted.

An *ATP7B* point mutation was created in HEK293T cells to mimic a WD relevant genotype and apply gene therapy. CRISPR/Cas9 vector PX459.ATP7B was transfected into HEK293T to induce an *ATP7B* KO. After single-cell separation, 93 cell clones were tested on Cu sensitivity in an MTT assay using a Cu concentration of 0.25 mM. About 82% of the tested cell clones showed an increased Cu sensitivity, suggesting an impaired Cu detoxification. Further analysis of cell viability in presence of different Cu concentrations confirmed previous results of increased Cu sensitivity (Fig 1A). At a Cu concentration of 0.5 mM, all of the tested cell clones showed no cell viability, whereas 24.7% of the HEK293T WT cells were vital. For sequence analysis 7 cell clones with decreased Cu resistance were cultivated and Sanger sequencing was performed. All analyzed cell clones indicated deletions in exon 2 of *ATP7B* (S2 Table). Cell clone #1 harbors a deletion of one cytosine nucleotide (p.E396KfsX11) of exon 2 (Fig 1B and S2 Table). This clone was named HEK293TΔC and used in the following for CRISPR/Cas9-mediated repair.

### Generation of *ATP7B* knockin by CRISPR/Cas9

Here, the genetic correction of an *ATP7B* point mutation using CRISPR/Cas9 technology was assessed. Previous repair experiments of the HEK293T *ATP7B* KO clone using the PX459.ATP7B plasmid and ssODNs showed neither an integration of the deleted cytosine (C) nucleotide, nor an integration of silent mutations or any Cas9 activity. Thus we assumed that the sgRNA of the PX459.ATP7B plasmid did not match to the protospacer region of the HEK293T *ATP7B* KO clone (Clone #1, HEK293TΔC cells) exhibiting a deletion of a C nucleotide. Using site-directed mutagenesis we deleted the C nucleotide of the PX459.ATP7B plasmid within the sgRNA and generated the PX459.ATP7BΔC plasmid for application in subsequent repair experiments.

**Fig 2:**
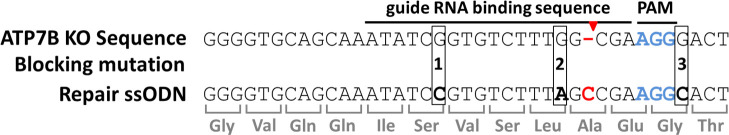
Scheme of CRISPR/Cas9-mediated *ATP7B* repair. *ATP7B* KO (HEK293TΔC) cells exhibit a point mutation in form of a cytosine nucleotide deletion (red hyphen). Cas9 cuts three nucleotides (red arrow) upstream the PAM region (blue). Repair ssODNs contain silent blocking mutations at positions 1, 2 and 3 (ssODN_3M) or at positions 2 and 3 (ssODN_2M).

**Fig 3:**
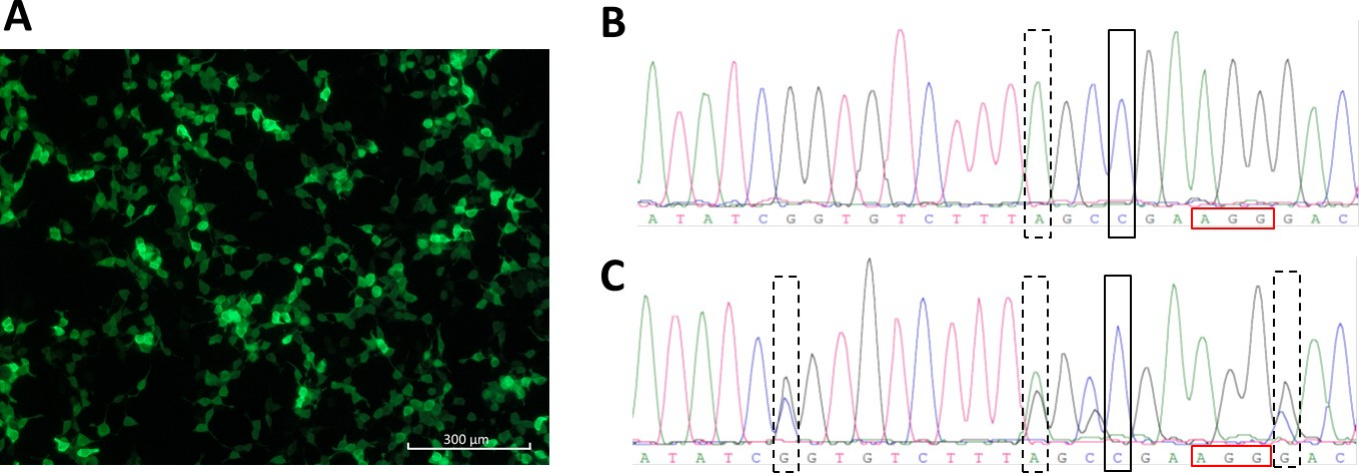
CRISPR/Cas9-mediated *ATP7B* repair. **(A)** HEK293TΔC cells were lipofected with pmaxGFP. GFP expression one day after lipofection is shown. Exemplary representation of Sanger sequence analyzes of **(B)** an *ATP7B* repaired homozygous HEK293TΔC cell clone (rectangle) carrying blocking mutation no. 2 (dashed rectangle) and of **(C)** an *ATP7B* repaired heterozygous HEK293TΔC cell clone (rectangle) carrying all three blocking mutations (dashed rectangles). PAM regions are framed with red rectangle.

HEK293TΔC cells were transfected using the PX459.ATP7BΔC plasmid plus a mixture of four different ssODNs that varies in total length (Fig 2 and S1 Table). In order to test the transfection efficiency and intensity, HEK293TΔC cells were transfected with a plasmid encoding GFP (pmaxGFP, Amaxa, Köln, Germany) and analyzed by fluorescence microscopy. 24 hours after lipofection an estimated cellular number of 90% displayed an intense GFP expression (Fig 3A). PX459.ATP7BΔC transfected cells were positively selected with 0.5 mM of Cu, 24 hours (day 1), or 72 hours (day 3) after lipofection, or with no Cu for a period of two days. Cells recovered two to three weeks after Cu treatment, and vital cells of every group underwent single-cell-cloning. Stable cell populations grew within three weeks after plating of the cells. A total of 126 cell clones were cultivated, of which 93 cell clones were analysed by Sanger sequencing. An overall CRISPR/Cas9 activity of 73% was calculated, including homozygous and heterozygous repaired cell clones, plus cell clones indicating deletions, which was independent of Cu or ssODN treatment (see sections below). Almost 50% of the cells indicated an *ATP7B* repair, of which 12% showed a homozygous repair (Fig 3B), and 37% a heterozygous *ATP7B* gene editing (Fig 3C).

### Copper selection post transfection enhances repair efficiency

**Fig 4:**
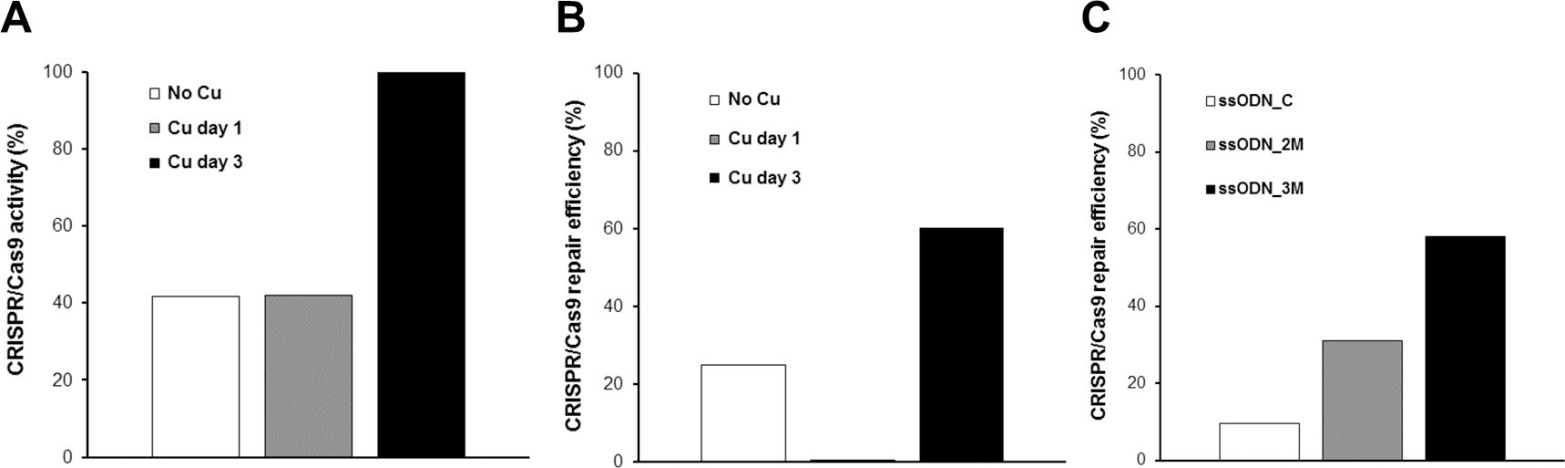
Activity and efficiency of CRISPR/Cas9 in *ATP7B* KI repair experiment. (**A**) Percentage of CRISPR/Cas9 activity in HEK293TΔC cells after Cu selection, comprising homozygous and heterozygous gene edited cell clones, and cell clones indicated sequence deletions. (**B**) Percentage of CRISPR/Cas9 repair efficiency in HEK293TΔC cells after Cu selection, comprising homozygous and heterozygous gene edited cells. **(C)** Percentage of CRISPR/Cas9 repair efficiency with regard to ssODN applications.

One of the aims of this study was to increase the efficiency of an *ATP7B* repair by the addition of Cu. Three groups of cells were transfected with plasmid PX459.ATP7BΔC plus ssODNs, and two of these groups were selected with 0.5 mM of Cu, one group received no Cu. The Cu selection that started 24 hours (day 1) after lipofection resulted in 41.9% CRISPR/Cas9 activity, which was comparable to the group that received no Cu (41.6%). Cu selection that started 72 hours (day 3) after lipofection led to an overall CRISPR/Cas9 activity of 100% (Fig 4A). These results show that a later start of cell selection using Cu leads to a higher yield of the CRISPR/Cas9 activity, based on heterozygous and homozygous clones and clones with deletions. Considering the CRISPR/Cas9 repair efficiency comprising heterozygous and homozygous repaired cell clones, a Cu selection three days after lipofection led to 60% of repaired clones, whereas a Cu selection one day after lipofection produced only cell clones exhibiting deletions (0% repair) (Fig 4B). Without any Cu selection, the repair efficiency was at least 25%, which may indicate that a selection with Cu at an early time point after lipofection reduces the yield of repaired clones.

### Assessment of ssODN quality

To identify the best conditions for a CRISPR/Cas9-mediated repair of a point mutation within the *ATP7B* gene, different ssODNs have been used in the current study. All three groups received a cocktail of ssODNs varying in total lengths of 61, 81, 101 and 121 nt, but differed in the number of silent or blocking mutations. The quality of the applied ssODNs was measured based on their repair efficiency. The repair efficiency of the control group exhibiting no blocking mutation (ssODN_C) was 9.5%. The group that received ssODNs with two blocking mutations (ssODN_2M) showed an efficiency of 31%, whereas the percentage of clones repaired with ssODNs carrying three blocking mutations (ssODN_3M) was 58%, indicating the most progressive attainment (Fig 4C). When ssODNs with a total of three silent mutations were used in a CRISPR/Cas9-mediated repair, a total of two mutations were most often inserted. When ssODNs carrying two silent mutations were used, only one mutation was most often inserted. We next addressed the question at which position a blocking mutation was most frequently integrated. All 18 clones being treated with ssODN_3M and undergoing homo- or heterozygous repair incorporated mutation No. 2. This blocking mutation is located two nucleotides upstream the repair site and within the guide RNA binding sequence (Fig 2). 16 out of 18 clones additionally integrated mutation No. 1, which is positioned 11 nucleotides upstream the repair site, and is located also within the guide sequence. 5 out of 18 clones additionally incorporated the blocking mutation No. 3, which is positioned seven nucleotides downstream the repair site, immediately behind the PAM region, but beyond the guide sequence. The group treated with ssODN_2M revealed 13 homo- or heterozygous repaired cell clones that all incorporated blocking mutation No. 2 and three out of 13 clones additionally integrated mutation No. 3. In summary, mutation No. 2 was the most frequent blocking mutation, which was inserted by ssODN_2M or ssODN_3M.

### CRISPR/Cas9-mediated *ATP7B* repair generates Cu resistance

**Fig 5:**
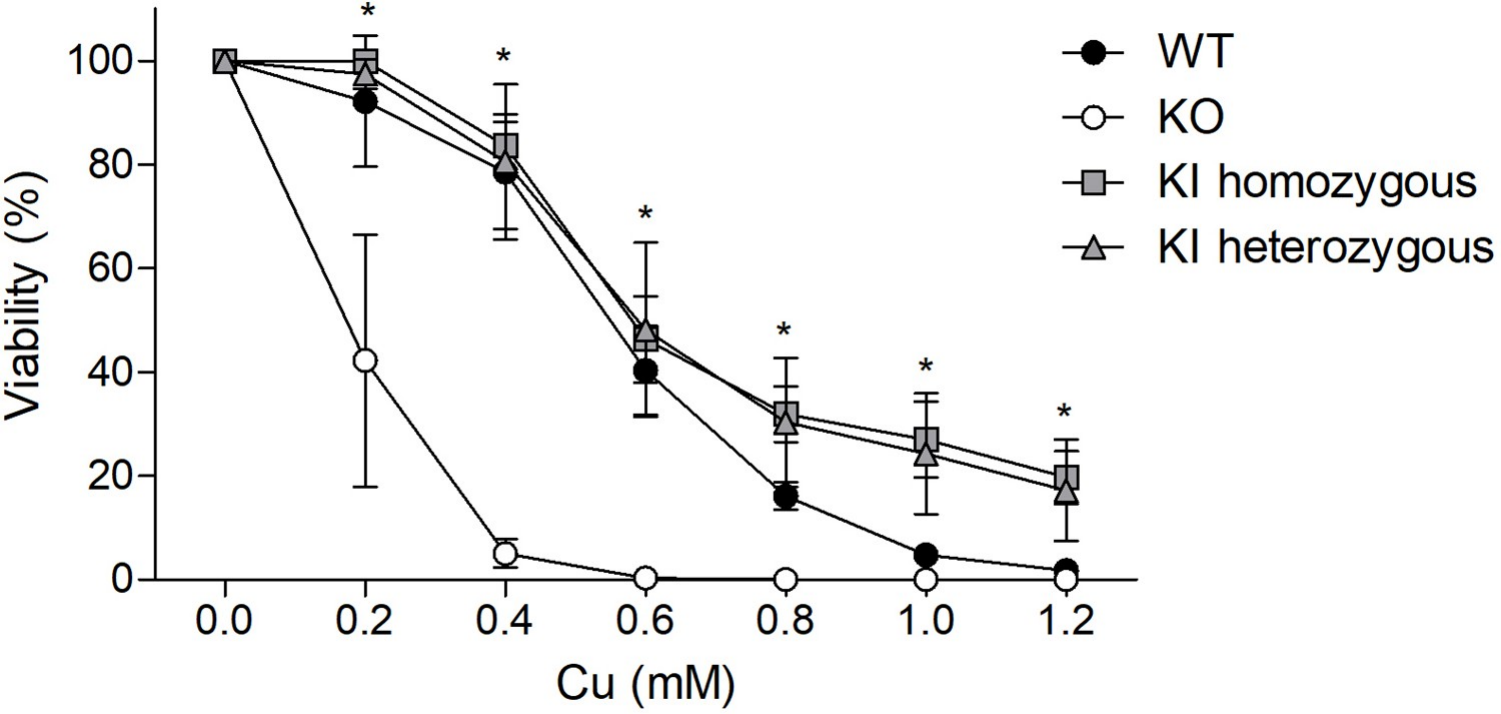
Cell viability of *ATP7B* KI cells after Cu exposure. HEK293T *ATP7B* WT cells (black circle), *ATP7B* KO cells (white circle), *ATP7B* KI homozygous (rectangle) and heterozygous (triangle) cell clones were cultivated for 48 hours using cell culture medium containing the indicated Cu concentrations. Cellular metabolic activities of cells were determined by MTT assay. The survival of cells is given as percentage relative to cells that received no Cu treatment (100%). Mean ± SE of four experiments is shown. * indicate significance (p < 0.05).

To evaluate the cellular Cu resistance after a CRISPR/Cas9-mediated repair of a point mutation within the *ATP7B* gene, we incubated the rescued cell clones to various Cu concentrations and measured the cell viability in an MTT assay (Fig 5). Four heterozygous repaired cell clones and four homozygous repaired cell clones were compared to parental HEK293TΔC cells and HEK293T *ATP7B* WT cells carrying an intact *ATP7B* gene. No differences in cellular viability were observed comparing the homo- and heterozygous repaired clones with HEK293T *ATP7B* WT cells, indicating a regained resistance to increasing Cu concentrations after CRISPR/Cas9 treatment. Cell viability of HEK293TΔC (KO) cells significantly differed to all other cell groups, ranging from 0.2–1.2 mM Cu. At a Cu concentration of 0.6 mM, HEK293TΔC KO cells showed no cell survival, whereas all *ATP7B* KI cells indicated a cell survival of 46–48%. Interestingly, no differences have been observed between homozygous and heterozygous *ATP7B* KI groups with regard to Cu sensitivity.

Since the *ATP7A* gene is a copper-transporting P-type ATPase mainly expressed in non-hepatic tissues, the expression level was analyzed by real-time RT PCR in HEK293T WT, KO and KI cells (S1 Fig). The results indicate no significant change within the *ATP7A* expression after induction of CRISPR/Cas9-mediated KO or KI of *ATP7B* as compared to WT HEK293T cells.

### ATP7B protein is restored after CRISPR/Cas9-mediated repair

**Fig 6:**
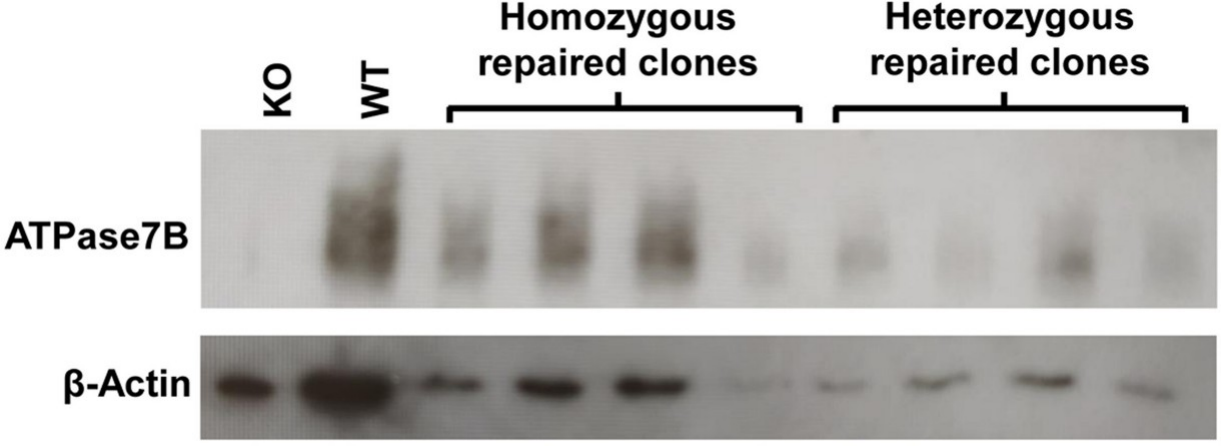
ATP7B protein expression after CRISPR/Cas9-mediated repair. Protein expression was analyzed by Western Blot analysis. ATP7B expression of four homozygous cell clones and four heterozygous cell clones were compared to HEK293T WT cells and *ATP7B* KO cells. β-Actin staining was used as a protein loading control.

In order to define the protein expression of ATP7B after CRISPR/Cas9-mediated repair a Western Blot analysis was performed, comparing HEK293T *ATP7B* WT cells with the HEK293TΔC (KO) cells, four homozygous repaired cell clones, and four heterozygous repaired cell clones (Fig 6). ATP7B protein expression was not detectable in HEK293TΔC cells, whereas ATP7B protein expression in all repaired homozygous cell clones was found to be as high as in HEK293T *ATP7B* WT cells, indicating a restoration of the ATP7B protein. Heterozygous cell clones showed a substantial protein expression, which was generally lower as compared to homozygous repaired cell clones, according to a lower protein synthesis by one repaired allele.

## Discussion

Since WD is characterized by mutations of the *ATP7B* Cu transporter new gene therapeutic treatment options are eligible and of high demand. This study is the first to demonstrate a gene correction of an *ATP7B* point mutation on human cellular level. First, using CRISPR/Cas9 gene editing, an *ATP7B* KO was successfully initiated in the human cell line HEK293T. After addition of Cu, KO cell clones showed a decreased cellular viability and indicated various deletions on exon 2, confirming a loss of function of ATPase7B. About 122 mutations on exon 2 have been registered so far, affecting the first four Cu binding sites of the *ATP7B* gene [39, 40]. Accordingly, the deletion of HEK293TΔC cells on exon 2 (c.1184delC) impairs the fourth Cu binding site. Interestingly, a naturally occurring mutation (c.1186G>T) was detected in the close proximity of this artificial mutation [41]. It is located between the PAM site and the cutting site of the current study, comprising a substitution (GAA>TAA), which leads to a stop codon. This homozygous point mutation was detected in six Egyptian children with WD that displayed neurological and/or hepatic manifestations. Since the CRISPR/Cas9-mediated point mutation of the current study (c.1184delC) also compromises the fourth Cu binding site it is conceivable that this mutation may have clinical relevance.

A gene correction of this point mutation was initiated using CRISPR/Cas9 technology and specific ssODNs. Previous experiments demonstrated that the repair of a point mutation cannot occur if the sgRNA sequence of the expressing plasmid does not fully match with the protospacer region of the target DNA. Apparently, if only one nucleotide is missing the base pairing and the following repair will not take place, demonstrating that the sgRNA used here is highly specific. We solved this problem by adapting the sgRNA of the plasmid to the C deletion of the HEK293TΔC cells using site-directed mutagenesis.

Combined with the appropriate selection method, in this case the addition of 0.5 mM of Cu three days after lipofection, a high yield of CRISPR/Cas9-mediated *ATP7B* repaired cell clones was achieved (60%). Moreover, the repair efficiency was enhanced using ssODNs that incorporated blocking mutations, which increase HDR accuracy by preventing re-cutting of the repaired allele by Cas9 nuclease [30]. The percentage of clones repaired with ssODNs carrying three blocking mutations (ssODN_3M) was 58%, indicating the most progressive attainment. Thus, the application of ssODNs carrying three blocking mutations, with at least two blocking mutations located within the guide RNA binding sequence, may represent to be a valuable tool for the repair of point mutations. In summary, it was shown that all 31 homo- and heterozygous repaired clones treated with either ssODN_3M or ssODN_2M have incorporated the blocking mutation at position No. 2, which is the closest to the repair site. Since mutation No. 3, which was located outside of the guide RNA sequence and was the least frequently installed mutation, one could argue that a blocking mutation at this position has no significant effect on the repair rate. Thus, the highest repair rate could be achieved if the blocking mutations were within the guide RNA region. However, since the highest repair rate was detected within the group with ssODN_3M, it could be assumed that the use of ssODNs carrying a total of three silent mutations is required in order to achieve a high CRISPR/Cas9-mediated repair rate.

In order to determine the function of the ATPase7B after the repair of an *ATP7B* point mutation, heterozygous and homozygous repaired cell clones were incubated in high Cu concentrations. Cellular viability of both repaired cell groups was as high as in HEK293T *ATP7B* WT cells, indicating a regain of Cu transporting function of ATPase7B. Interestingly, there was no difference observed between both groups, demonstrating that even a heterozygous repair of a point mutation on one allele leads to resistance to high Cu concentrations. This may go along with the fact that *ATP7B* heterozygous patients present no or mild clinical symptoms [42].

*ATP7A* gene is a copper-transporting P-type ATPase and mainly expressed in non-hepatic tissues such as kidneys. Therefore, one could assume that *ATP7A* may compensate a high and toxic Cu concentration in HEK293T cells, when *ATP7B* is knocked out. However, since the *ATP7A* expression in all 3 cell lines (WT, KO and KI) is in the same range, it can be assumed that *ATP7A* has no effect on the compensation of the toxic copper. Moreover, it also proves that the CRISPR/Cas9 treatment does not affect the *ATP7A* expression.

Since WD is characterized by multiple different forms of heterozygous, homozygous and compound heterozygous mutations, ranging from point mutations, insertions or deletions, a potential therapeutic treatment using CRISPR/Cas9 technology has to be individualized for every WD patient before administration. Moreover, delivery of therapeutic CRISPR/Cas9 molecules to the liver has to be guaranteed. Investigations on liver-specific targeting of the CRISPR/Cas9 system have been demonstrated by e.g. Singh et al., using adeno-associated virus (AAV) 9-delivery of truncated guide RNAs and Cas9 under the control of a computationally designed hepatocyte-specific promoter, leading to liver-specific and sequence-specific targeting in the mouse factor IX (F9) gene [43]. Compared to the use of viral vectors for gene therapy, which may activate the innate or adaptive immune system and leads to severe inflammatory response, CRISPR/Cas9 application may also be used to circumvent these issues by non-viral application, thus representing the safer therapy option. Since the liver has the advantage of being a target organ for oligonucleotide therapeutics [44, 45], a systemic application of naked plasmid DNA offers the opportunity for a high yield of gene editing in monogenetic liver diseases. In animal models, this was accomplished by hydrodynamic delivery, which is an effective non-viral method of liver-targeted gene delivery via blood circulation [46–48]. Once in the liver, the pressurized solution enlarges the liver fenestrae, and forces the permeability of the plasma membrane to allow the DNA to enter the cells [49]. Several studies combined this method with the application of CRISPR/Cas9 therapeutic molecules to treat rare liver diseases [50–52]. In a mouse model of the human disease hereditary tyrosinemia Yin et al. demonstrated a CRISPR/Cas9-mediated correction of the Fah mutation in hepatocytes [53]. The hydrodynamic injection of components of the CRISPR/Cas9 system resulted in an expression of the wild-type Fah protein in ~1/250 liver cells and rescued the body weight loss phenotype. The same group developed an optimal set for a safer clinical application by using chemically modified RNAs [54]. These studies underline the potential of the CRISPR/Cas9 system for allele-specific genome editing in WD. Since WD livers exhibit high concentrations of Cu, a CRISPR/Cas9-mediated repair of the *ATP7B* gene may benefit from this condition as a selection advantage.

Lately, CRISPR/Cas9 engineered T cells were applied in patients with refractory cancer, demonstrating the feasibility of CRISPR gene editing for cancer immunotherapy [55]. Although there are still limitations of CRISPR/Cas9 application in the clinic, e.g. *in vivo* off-target effects, or possible immune responses to the Cas9 protein, which is a bacterial enzyme, this technology may be a first step towards curing WD. The current study demonstrates that CRISPR/Cas9 technology is not only highly efficient in introducing specific *ATP7B* mutations, but also in correcting *ATP7B* point mutations, which are highly frequent in WD patients. While the use of ssODNs is limited to non-viral delivery methods, the application of these therapeutic molecules may initiate a direct and safe correction of point mutations within the *ATP7B* gene, thus contributing to a WD gene modification with high therapeutic potential in clinical application.

## Supporting information

S1 Raw image Original Western Blot image. ATP7B protein expression of four homozygous cell clones and four heterozygous cell clones were compared to HEK293T WT cells and *ATP7B* KO cells. β-Actin staining was used as a protein loading control. Panels 3 to 12 were used to create Fig 6. Molecular weight marker obtained from Thermoscientific Fermentas (#SM1811). Method used to capture image was by smartphone camera Xiaomi Mi A3.
(TIF)

S1 Fig Expression of *ATP7A* mRNA. Real-time RT-qPCR analysis of WT, KI homozygous, KI heterozygous and KO HEK293T cells. Values were normalized to *GAPDH* house-keeping gene and *ATP7A* expression of HepG2 cells (ΔΔCt method). Mean ± SD are shown (n = 2–3).
(TIF)

S1 Table List of single-stranded oligo DNA nucleotides (ssODNs). All 12 ssODNs carry the reintroduced cytosine nucleotide (red), and the PAM region (blue). Group ssODN_3M (1–4) carry three blocking mutations, group ssODN_2M (5–8) carry two blocking mutations, shown as capital letters. Group ssODN_C (9–12) carry no blocking mutations.
(TIF)

S2 Table List of cell clones after CRISPR/Cas9-mediated *ATP7B* KO. Lengths of nucleotide deletions, cutting sites and genotypes are depicted. PAM sequence is indicated in bold.
(TIF)

## Abbreviations

Cu: copper
KO: *ATP7B* knockout
KI: *ATP7B* knockin
WD: Wilson disease
ssODN: single-stranded oligo DNA nucleotide
CRISPR/Cas9: clustered regularly interspaced short palindromic repeats (CRISPR) associated nuclease 9
