# Supplementary figures and images for: CRISPR/Cas9-mediated correction of mutated copper transporter ATP7B
Source: PLoS One. 2020 Sep 30;15(9):e0239411. doi: 10.1371/journal.pone.0239411 (PMC7526882; doi:10.1371/journal.pone.0239411)

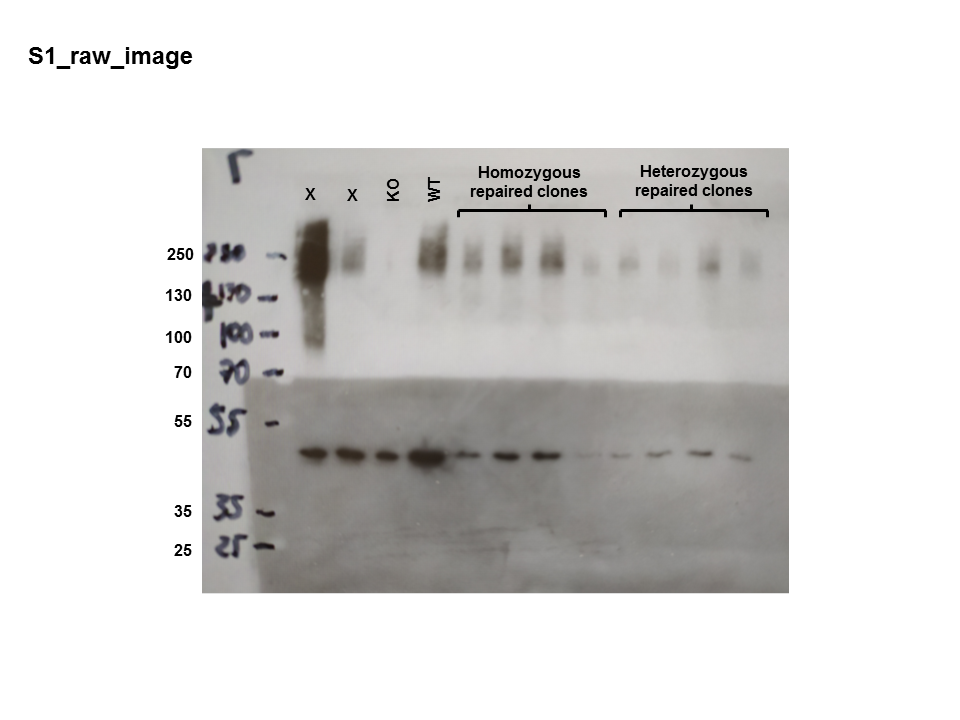

Supplement: S1 Raw image — ATP7B protein expression of four homozygous cell clones and four heterozygous cell clones were compared to HEK293T WT cells and ATP7B KO cells. β-Actin staining was used as a protein loading control. Panels 3 to 12 were used to create Fig 6. Molecular weight marker obtained from Thermoscientific Fermentas (#SM1811). Method used to capture image was by smartphone camera Xiaomi Mi A3. (TIF) [file pone.0239411.s001.tif]

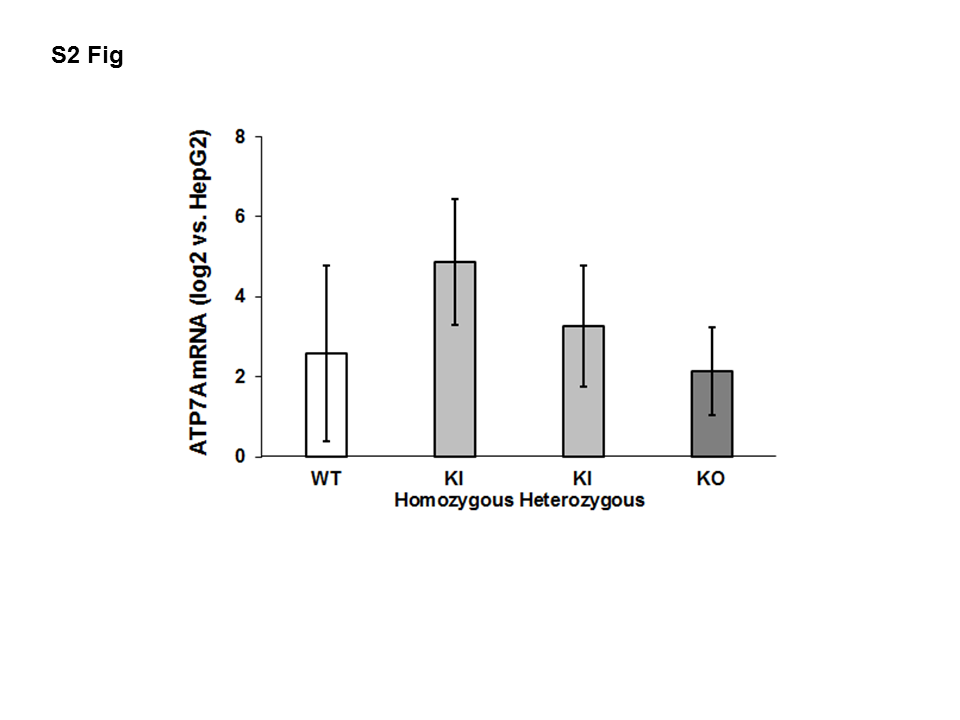

Supplement: S1 Fig — Real-time RT-qPCR analysis of WT, KI homozygous, KI heterozygous and KO HEK293T cells. Values were normalized to GAPDH house-keeping gene and ATP7A expression of HepG2 cells (ΔΔCt method). Mean ± SD are shown (n = 2–3). (TIF) [file pone.0239411.s002.tif]

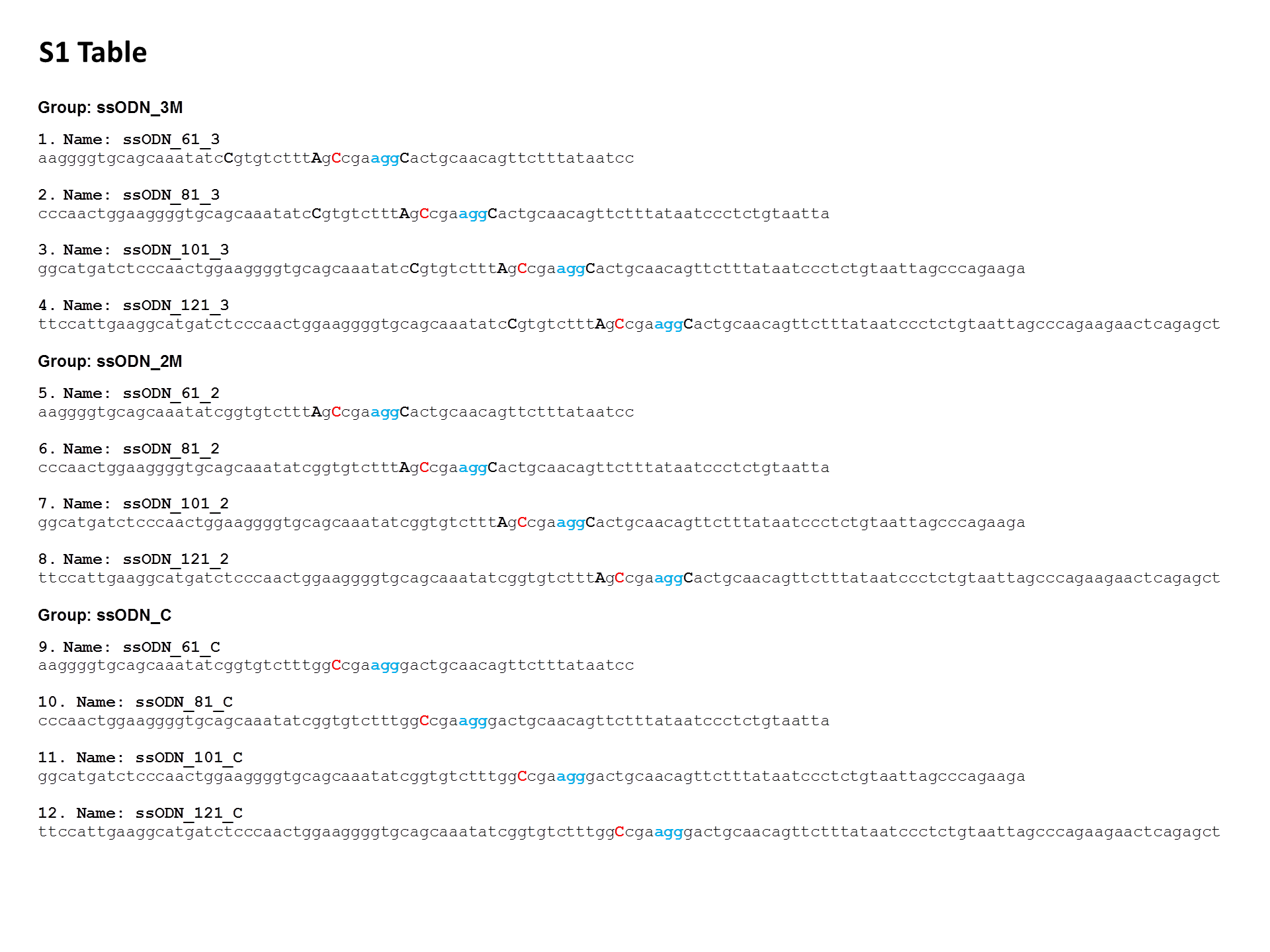

Supplement: S1 Table — All 12 ssODNs carry the reintroduced cytosine nucleotide (red), and the PAM region (blue). Group ssODN_3M (1–4) carry three blocking mutations, group ssODN_2M (5–8) carry two blocking mutations, shown as capital letters. Group ssODN_C (9–12) carry no blocking mutations. (TIF) [file pone.0239411.s003.tif]

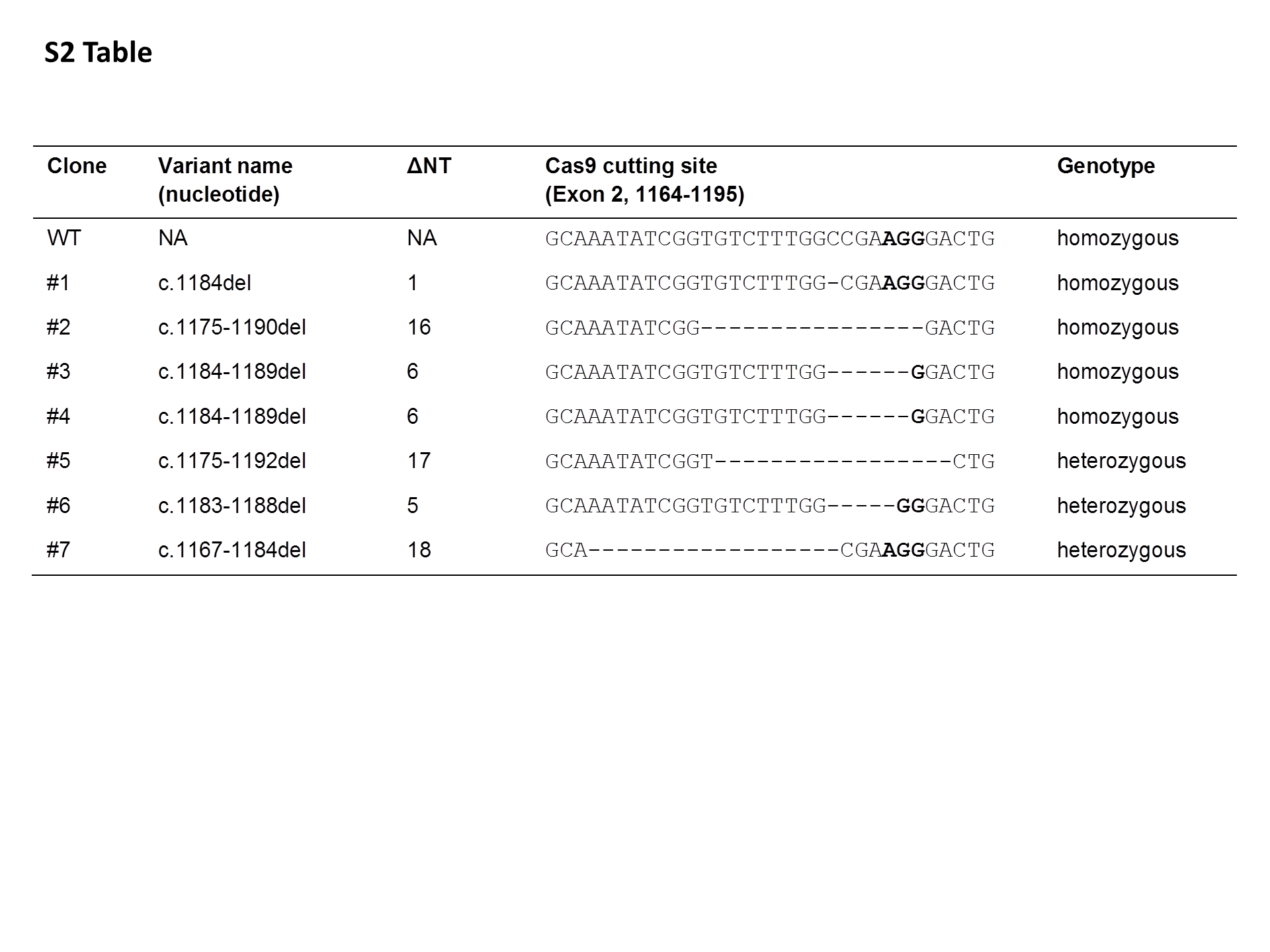

Supplement: S2 Table — Lengths of nucleotide deletions, cutting sites and genotypes are depicted. PAM sequence is indicated in bold. (TIF) [file pone.0239411.s004.tif]
